# 2120. Respiratory Viral Infections in Patients with Hematologic Malignancies

**DOI:** 10.1093/ofid/ofac492.1741

**Published:** 2022-12-15

**Authors:** Jorge Luis Cardenas, Mohammed Raja, Jose F Camargo, Michele I Morris, Yoichiro Natori

**Affiliations:** University of Miami Miller School of Medicine, Coral Gables, Florida; University of Miami Miller School of Medicine and Miami Transplant Institute, Jackson Health System, MIAMI, Florida; University of Miami Miller School of Medicine and Miami Transplant Institute, Miami, Florida; University of Miami Miller School of Medicine and Miami Transplant Institute, Miami, Florida; University of Miami Miller School of Medicine and Miami Transplant Institute, Jackson Health System, MIAMI, Florida

## Abstract

**Background:**

Respiratory virus infections (RVI) are an important cause of pneumonia with increased risk of morbidity and mortality in patients with hematologic malignancies (HM) especially after stem cell transplantation. However, data on RVI in this population without stem cell transplant is still lacking. Here we aim to look at trends of RVI in HM population.

**Methods:**

We conducted a retrospective cohort study of adult patients with HM diagnosed with RVI at our institution. We analyzed data from multiplex qualitative PCR-based respiratory viral panel (RVP) samples taken from January 2016 to June 2020. Lower respiratory tract infection (LRTI) was defined as abnormal chest radiography with positive RVP.

**Results:**

Fifty-seven unique episodes were analyzed out of 54 patients. Thirty (52.6%) were females and the most common underlying malignancy was Non-Hodgkin’s Lymphoma (21, 36.8%). Median age was 61 (range 23-92) years old and 44/57 (77.1%) patients received chemotherapy within 30 days prior to RVI. Patient characteristics are summarized in Table 1. Most common viruses included Rhinovirus/Enterovirus (25, 43.8%), Influenza A/B (14, 24.5%) and RSV A/B (7,12.2%), respectively. Out of 39 patients who underwent chest imaging, 13/39 (33.3%) had evidence of LRTI. Out of 45 cases diagnosed as outpatients, 11/45 (24.4%) required hospital admission. ICU admission and mechanical ventilation were required in 5/57 (8.7%) and 2/57 (3.5%), respectively. Bacterial and fungal co-infection were seen in 1 (Pseudomonas spp) and 3 (Aspergillus spp.), respectively. Of note, only 1/57(1.8%) died at 30 days after RVI diagnosis.

**Figure:**
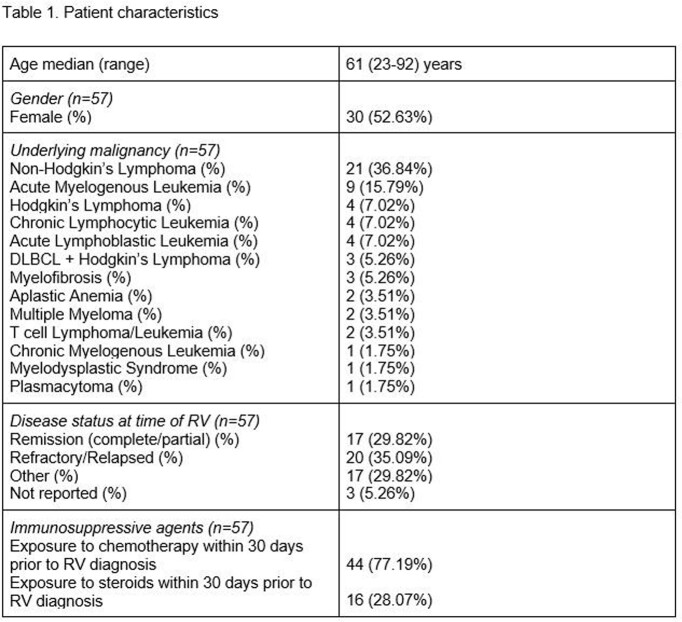

**Conclusion:**

Only one third developed LRTI after RVI in HM patients with very low 30-day mortality. However, we still found several bacterial and fungal co-infections. Thus, adequate diagnosis for RVI and co-infections with proper treatment should be required. Further studies with larger sample size are still needed.

**Disclosures:**

**All Authors**: No reported disclosures.

